# Measurement of the Entangled Two‐Photon Absorption Cross‐Sections of 4‐Aminoazobenzene

**DOI:** 10.1002/cphc.70403

**Published:** 2026-05-11

**Authors:** Sena Hashimoto, Hisaki Oka, Izumi Iwakura, Tomoyuki Horikiri

**Affiliations:** ^1^ Kanagawa University Yokohama Japan; ^2^ Society for the Promotion of Science (JSPS) Tokyo Japan; ^3^ Yokohama National University Yokohama Japan

**Keywords:** azobenzene derivative, entangled photons, two‐photon absorption

## Abstract

Recently, entangled two‐photon absorption (ETPA) has attracted considerable attention, yet reported cross‐sections vary widely. In this study, we focused on 4‐aminoazobenzene, an azobenzene derivative that exhibits extremely low one‐photon absorption in the wavelength range of time‐entangled photon pairs. By measuring the time correlation between the two photons transmitted through the sample solution and separating correlated from uncorrelated counts, we distinguished between ETPA and linear‐loss contributions. The ETPA cross‐section is only ≈10^7^‐fold lower than the one‐photon absorption cross‐section and ≈10^18^‐fold higher than the classical TPA cross‐section (evaluated under classical two‐photon excitation at a photon‐flux density of ≈10^8^ photons·s^–1^·cm^–2^). Moreover, ETPA is measurable at a photon‐flux density of ≈10^8^ photons·s^–1^·cm^–2^.

## Graphical abstract

Using 4‐aminoazobenzene with extremely low one‐photon attenuation in the EP band, we isolated entangled two‐photon absorption from linear loss by separating correlated and uncorrelated photon counts. The resulting σ_E_ is ≈10^18^ larger than δ^(2)^ (at ≈10^8^ photons·s^–1^·cm^–2^), while remaining ≈10^7^ smaller than δ^(1)^.

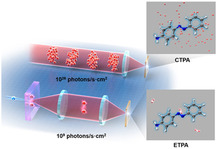

## 1 Introduction

Two‐photon absorption (TPA) is a nonlinear optical process in which two photons are simultaneously absorbed by a single molecule and therefore occurs near the focal spot, where the photon‐flux density is extremely high; this differs from one‐photon absorption, which proceeds over the illuminated area. Since extremely high photon‐flux density directly results in spatially high resolution, TPA processes are applied in the fields of bioimaging and microscopic spectroscopy [1, 2]. Another potential application of TPA is the activation of optical switching molecules in vivo. Azo compounds are examples of optical switching molecules [3, 4, 5, 6] and have been extensively investigated due to their rapid changes in absorption wavelength, refractive index, and polarity; these changes originate from their *trans‐cis* photoisomerization. Generally, efficient photoisomerization of azo compounds requires excitation using visible and ultraviolet wavelengths [6, 7, 8]. However, for use as an in vivo optical switching molecule, ultraviolet and visible light are absorbed or scattered by living tissues and therefore cause direct optical damage and difficulty in the deep penetration of living tissues [9]. TPA using the first near‐infrared window (NIR‐I; 700–900 nm) has high permeability in living tissues, can resolve these problems, and has attracted much attention [10, 11, 12]. In TPA via NIR‐I, spatially focused and high‐power femtosecond laser pulses are used [13]. For example, femtosecond laser pulses with a photon‐flux density of 10^27^ – 10^29^ photons·s^–1^·cm^–2^ can be used for TPA measurements of small organic compounds [14, 15, 16, 17]. However, these laser pulses with extremely high photon‐flux density often lead to damage to living tissues due to photothermal effects [18, 19]; therefore, efficient TPA with low photon‐flux density is needed.

In the field of optics, various methods are considered to increase the efficiency of TPA using NIR‐I rather than conventional femtosecond laser pulses. For example, by using thermal light (incoherent light), the two‐photon excitation fluorescence intensity can be improved twofold compared to that obtained by using coherent light (laser light) [20]. Furthermore, methods to utilize squeezed light [21, 22, 23, 24] and an entangled‐photon pair (EPP) with intrinsic simultaneity of two photons [25, 26, 27, 28, 29, 30, 31, 32, 33, 34, 35, 36] have also been reported. In two‐photon excitation, the time coincidence of two photons is quite important because an atom or a molecule needs to absorb two photons simultaneously. Since squeezed light and EPP exhibit time coincidence, which is absent in classical light, efficient two‐photon excitation can be expected. In fact, an enhanced transition rate has been theoretically predicted [25, 28] and experimentally observed [22] for narrowband (long‐pulse) squeezed light at low photon fluxes in atoms and solids. Experimentally, since the first measurement of entangled two‐photon absorption (ETPA) in organic compounds was reported in 2006 by Goodson et al. [30], various measurements have been reported. TPA has been measured in organic compounds using EPP, and photon‐flux densities as low as the photon‐counting level (≈10^10^ photon·s^–1^·cm^–2^) have been attained [31, 33]. Even in organic compounds, TPA using EPP has been theoretically predicted to enhance the efficiency of TPA compared with that of conventional femtosecond pulse lasers [21, 37, 38, 39, 40]. Recently, interest in the ETPA of organic compounds has increased; however, despite measurements of the same compound using similar techniques, considerable discrepancies have been observed among experimental results, depending on the concentration. The cause of this discrepancy was reported in 2022 by U’Ren et al. to be the linear loss within the EPP wavelength range [41]. Afterwards, the effects of linear loss (stationary absorption and scattering) [21, 41, 42, 43, 44, 45] were discussed. Because linear loss and other background processes can mimic an apparent ETPA signal, additional strategies to certify ETPA have been proposed [46].

In this study, we focused on 4‐aminoazobenzene (4‐AmAB); this is a small organic compound with extremely low one‐photon absorption and light scattering in the EPP wavelength range (750–850 nm). The details of sample selection are described in Section S1 of the Supporting Information. Furthermore, using EPPs generated via spontaneous parametric down‐conversion (SPDC) with a narrowband continuous‐wave (CW) pump light, we measured the time correlation between the two photons transmitted through the sample solution and separated correlated counts from uncorrelated counts. This allowed us to distinguish between ETPA and linear‐loss contributions and to evaluate the ETPA cross‐section, $\sigma_{E}$, of 4‐AmAB.

## 2 Results and Discussion

### 2.1 Theory: ETPA Cross‐Section

ETPA is a TPA process driven by EPPs that exhibit temporal and energy correlations. EPPs generated via SPDC under energy‐conservation and phase‐matching conditions exhibit strong quantum correlations; therefore, detection of one photon can herald the presence of its partner photon subject to the same constraints [13]. Owing to these correlations, the ETPA rate per molecule, $r_{ETPA}$ [photon pairs·s^–1^·cm^–2^], depends linearly on the incident EPP flux density at the sample, ϕ [photon pairs·s^–1^·cm^–2^], in contrast to classical two‐photon absorption (CTPA), for which the absorption rate, $r_{CTPA},$ scales with the square of the incident photon‐flux density, $\phi^{\prime }$ [photons·s^–1^·cm^–2^]. Based on previous research [33, 41], $\sigma_{E}[{\mathrm{cm}}^{2}\cdot {\text{molecule}}^{-1}],$ was estimated by assuming that $r_{ETPA}$ is proportional to ϕ, as

(1) $$r_{ETPA}=\sigma_{E}\cdot \phi$$

where ϕ is estimated from the measured two‐photon coincidence count rate (i.e., the detected photon‐pair rate) of the EPPs, 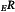 [photon pairs·s^–1^].

In the evaluation of $\sigma_{E}$ using the optical arrangement of transmission, we first measure the two‐photon coincidence count rates of EPP transmitted through the solvent and the sample solution, 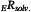 and 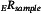. The absorbed photon‐pair count rate of EPP 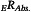 is evaluated from their difference after subtracting contributions from linear losses (LL):




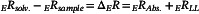






(2)

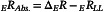

where 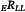 represents LL not attributable to ETPA (e.g., losses in the solvent, sample cell, and optical components). The measurement of 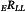 is described in the section “Measurement of Linear Loss”. Using 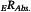, $r_{ETPA}$ is written as



(3)

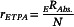

where N[molecule] is the number of solute molecules in the interaction volume. N is calculated from Avogadro's number $N_{A}[\text{molecule}\cdot {\mathrm{mol}}^{-1}]$, the molar concentration of the sample solution, $c[\mathrm{mol}\cdot L^{-1}]$, and the interaction volumeV[L] as

(4) $$N=c\cdot V\cdot N_{A}$$

Here, V is evaluated from the focusing condition and the optical path‐length of the cell (interaction length), using the same focusing approximation described below for A. From Equations (1), (3) and (4)),



(5)

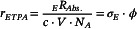




The ϕ of the EPPs that can interact with the solute molecules is estimated by dividing 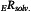 by the effective focusing area $A[{\mathrm{cm}}^{2}]$:



(6)

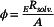




When the optical path length of the sample is less than twice the Rayleigh length, the effective focusing area A is approximated as twice the area of the focal spot. Combining Equations (5) and (6), $\sigma_{E}$ can be written as



(7)

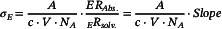




The proportionality constant *Slope* is obtained from the linear relationship between 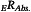 and 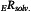 (e.g., the slope of a linear fit). When the intercept is negligible, it is equivalently expressed as



(8)

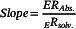




Because 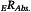 and 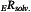 are acquired with the same detection system under identical settings, any constant overall coincidence (pair) detection efficiency is common to both measurements and cancels in the ratio, and hence in Slope.

### 2.2 Construction of the ETPA Measurement System

**FIGURE 1:**
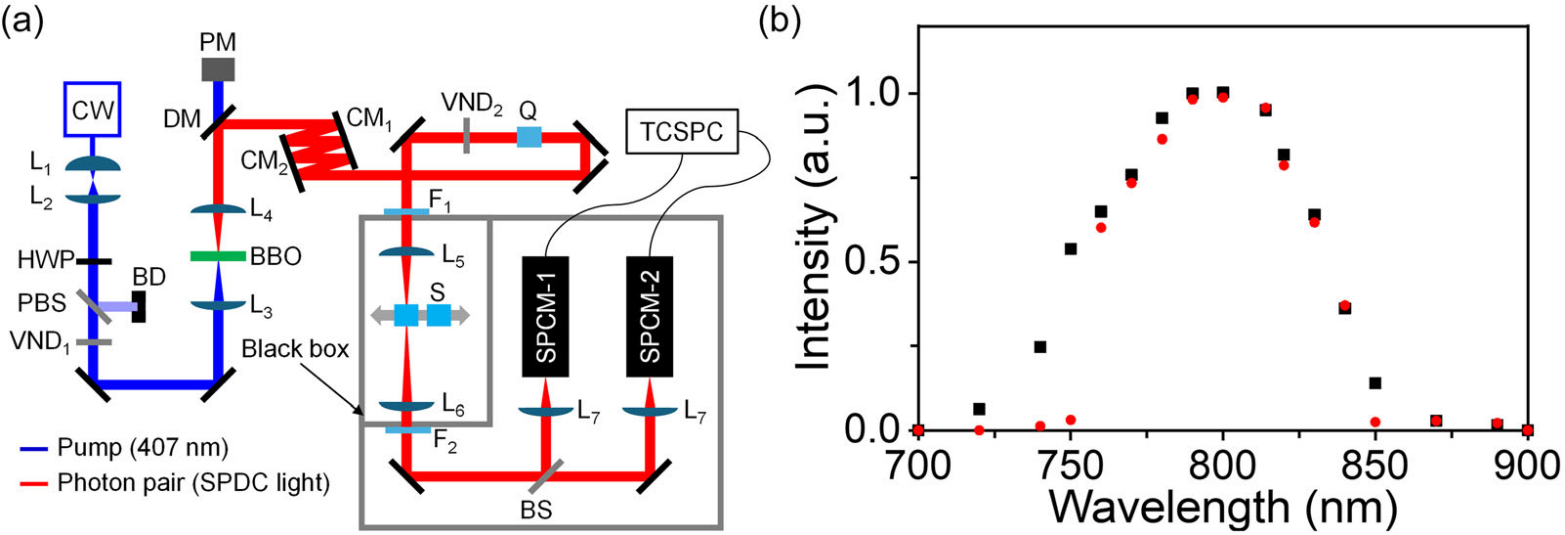
ETPA measurement system. (a) Schematic figure and (b) EPP spectra measured in front of F_1_ (black dot) and behind F_1_ (red dot).

The ETPA measurement system (Figure 1a) used a 407 nm CW laser as the SPDC pump and the SPDC pump intensity was adjusted by controlling the transmittance of a variable neutral density filter (VND_1_). EPPs were generated via SPDC in a nonlinear crystal (β‐BaB_2_O_4_, BBO, Type‐I). The generated EPPs were reflected by a dichroic mirror (DM). The residual 407 nm light that was not converted by SPDC and was transmitted through the DM was measured using a power meter. The SPDC pump light intensity incident on the BBO was estimated by correcting for losses due to transmission through the optical components (BBO, L_4_, DM) using the actual measured transmittance. Dispersion was further compensated using a chirped mirror pair (CM) and a quartz block (Q).

After the 407‐nm light passed through the BBO, the optical axis of the residual pump light coincided with that of the EPP. The residual pump light was suppressed by ~20 orders of magnitude using a combination of DMs and filters (F_1_; two 750 nm long‐pass filters and one 850‐nm short‐pass filter), reducing it to below the detection limit of the single‐photon counters (SPCM). The EPP entering the black box was focused into a 1 mm path‐length sample cell (S) using a convex lens (L_5_) and the transmitted light was collimated with another convex lens (L_6_). The filters (F_2_; same passband as F_1_) were then inserted to block light from the sample, such as fluorescence, whose wavelength range differs from that of the EPP. After the transmitted light was divided by a beam splitter (BS) into two beams at a 1:1 intensity ratio, each beam was focused onto an SPCM. A time‐correlated single‐photon counting board was used to measure the two‐photon coincidence counts, *C* [two‐photons], i.e., the number of detected two‐photon events [47]. The time difference between detections at SPCM‐1 and SPCM‐2 was defined as the delay time (*τ*). The number of single photons detected by SPCM‐2 at each delay time (*Cτ*) was measured. The EPP intensity was adjusted by rotating VND_2_. The focused area on the sample (A) was 2.9 × 10^–5^ cm^2^; additionally, by considering the optical path‐length (0.1 cm), the interaction volume (V) was calculated to be 2.9 × 10^–6^ cm^3^. For example, ϕ at the focal spot was 1.5 × 10^8^ [photon pairs·s^–1^·cm^–2^] when the SPDC pump light intensity was 34 μW. The EPP spectra (Figure 1b) were measured by recording *C* while scanning the monochromator wavelength for the light incident on SPCM‐2. Further experimental details are provided in Section S2 of the Supporting Information.

### 2.3 Sample Preparation

**FIGURE 2:**
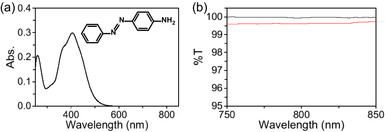
(a) Stationary absorption spectrum (1 mm path‐length) of 4‐AmAB in a DMSO solution using a UV–visible spectrophotometer. The concentration of the solution was 1 × 10^–4^ mol·L^–1^. (b) Stationary transmittance spectra (1 mm path‐length) of 4‐AmAB in DMSO solution. The concentration of the solution for the black line was 1 mol·L^–1^, and that for the red line was 5 mol·L^–1^.

All solutions were stored in screw‐capped silica‐quartz cells (1 mm path‐length) for the stationary absorption, CTPA, and ETPA measurements. The UV–visible absorption spectrum of 4‐AmAB in DMSO solution (1 × 10^–4^ mol·L^–1^) was measured (Figure 2a). 4‐AmAB exhibited absorption bands at approximately 400 nm, with an absorption maximum at 407 nm. A symmetry‐allowed π– π* transition gave rise to a broad absorption band extending from 350 to 500 nm, whereas a symmetry‐forbidden n– π* transition was observed on the longer‐wavelength side with very weak intensity. In the constructed ETPA measurement system, the SPDC pump wavelength was 407 nm, and the energy of a 407 nm photon equals the sum of the energies of the EPP. Therefore, 4‐AmAB can be excited via TPA induced by the EPP generated in this system. In addition, stationary transmittance spectra of the DMSO solutions at the two concentrations (1 and 5 mol·L^–1^), relative to solvent DMSO, were measured within the EPP wavelength range (750–850 nm) (Figure 2b). Both solutions used for ETPA measurements showed a transmittance difference of less than 0.35% relative to solvent DMSO over 750–850 nm. These results indicated that one‐photon absorption in the EPP wavelength region is extremely low (<0.35% attenuation even at the highest concentrations) and that neither a long‐wavelength absorption tail nor solute aggregation at high concentrations contributes appreciably in this range.

### 2.4 Measurement of _*T*_ *R*

As shown in Equation (1), $r_{ETPA}$ depends linearly on ϕ [photon pairs·s^–1^·cm^–2^], whereas $r_{CTPA}$ depends on the square of $\phi^{\prime }$ [photons·s^–1^·cm^–2^]. Since a photon pair consists of two photons, $2\phi =\phi^{\prime }$. Thus, ETPA and CTPA exhibit different relationships between the absorption rate (*r*) and photon‐flux density ($\phi^{\prime }$). Accordingly, ETPA and CTPA were differentiated by evaluating the $\phi^{\prime }$‐dependence of *r*. The SPDC pump light intensity is linearly proportional to $\phi (\phi^{\prime })$; thus, $\phi (\phi^{\prime })$ can be tuned by changing the SPDC pump light intensity. The SPDC pump light intensity was varied in 11 steps from 34 μW to 3.1 mW, and *Cτ* values were measured at each step in ascending order of pump light intensity. For each sample, the measurement was repeated three times, and the average of the results was used. The integration time for each measurement was 100 s. *Cτ* was recorded over the 0–100 ns delay‐time window. For the evaluation of *C*, only the 0–46 ns interval was used, as described in the “EPP detection efficiency” section. Accordingly, *C* was calculated by integrating *Cτ* from 0 to 46 ns using Equation (9) .

(9) $$C=\int_{0}^{46}C_{\tau }$$

*C* was integrated over 100 s, and the total count rate per second (_*T*_ *R* [two‐photons·s^–1^]) was calculated as follows:



(10)

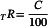




The measured total count rate _*T*_ *R* includes _*E*_ *R*, _*A*_ *R* (accidental count rates [two‐photons·s^–1^]), and _*N*_ *R* (noise count rates [two‐photons·s^–1^]). _*A*_ *R* originates from uncorrelated two‐photon events, and _*N*_ *R* includes contributions from environmental noise and dark counts. _*T*_ *R* is defined as



(11)

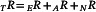

where *n* counts of _*E*_ *R* correspond to *n* photon pairs, whereas *n* counts of _*A*_ *R* and _*N*_ *R* correspond to 2*n* photons.

### 2.5 Measurement of _*N*_ *R*

**FIGURE 3:**
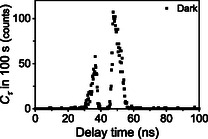
Results from the coincidence count rates derived from environmental noise.

In this system (Figure 1a), _*N*_ *R* was measured using a cell containing the solvent DMSO with the laser light turned off (Figure 3). When the laser light is turned off, the measured _*T*_ *R* value equals the _*N*_ *R* value. In Figure 3, the horizontal axis represents the delay time, and the vertical axis represents *Cτ* at each delay time. *C* was obtained by integrating the *Cτ* values shown in Figure 3 from 0 to 46 ns using Equation (9). Using the obtained *C* (790 [two‐photons·100 s^–1^]), _*T*_ *R* was calculated with Equation (10) ; under these conditions, _*T*_ *R=*_*N*_ *R=*8 [two‐photons·s^–1^].

By subtracting the histogram obtained when the pump light was turned off (_*N*_ *R*) from each measured histogram (_*T*_ *R*), we extracted the count rates originating from photons generated via SPDC (hereafter referred to as SPDC photons), i.e., _*E*_ *R*+_*A*_ *R*. To avoid the influence of environmental noise caused by the opening and closing of the black box, two cells (for the solvent and the sample solution) were set up as a pair on an electric stage (stage). This configuration allowed the samples to be exchanged while the black box remained closed, enabling detection of transmission‐rate differences as small as 0.1%.

### 2.6 Confirmation of the Optical Paths of the Photons Detected by SPCM‐1 and SPCM‐2

When the laser light was turned on and a shield was inserted at the position of the sample cell, the _*T*_ *R* value decreased to a level nearly equal to the _*N*_ *R* value. Therefore, the photons detected by SPCM‐1 and SPCM‐2 originated from light transmitted through the sample solution and propagated along the same optical path as the EPP.

### 2.7 Influence of the SPDC Pump Light (407‐nm Photons) on _*T*_ *R*

By using three interference filters as the black box windows to transmit the EPP wavelength, we confirmed that the 407‐nm photons were effectively blocked, as described below. A BBO crystal is a nonlinear crystal in which SPDC occurs via angular phase matching. When the BBO crystal was rotated around the optical axis of the pump light to an angle at which SPDC did not occur, the _*T*_ *R* value was nearly equal to the _*N*_ *R* value. Similarly, when the BBO crystal was removed from the optical path, the _*T*_ *R* value was nearly equal to the _*N*_ *R* value. Thus, under the conditions where SPDC did not occur, _*T*_ *R* did not change even when the SPDC pump light was turned on or off, confirming that _*T*_ *R* was not affected by the 407‐nm photons.

These results indicate that _*A*_ *R* primarily originated from single‐photon detections arising from EPPs in which one photon was lost along the optical path (e.g., by reflection or scattering at optical components such as lenses and filters, or by absorption or scattering in the sample, including the solvent and the cell), and that _*A*_ *R* was not caused by the count rates from the residual pump light. Hereafter, such a photon is referred to as an EPP‐derived unpaired photon (EUP). Thus, SPDC photons consist of intact EPPs and EUPs.

### 2.8 EPP Detection Efficiency

**FIGURE 4:**
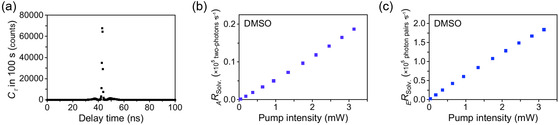
(a) Results from the coincidence counting measurement for the EPP transmitted through the cell containing DMSO at an SPDC pump intensity of 34 μW. (b) _*A*_ *R*_solv._ and (c) _*E*_ *R*_solv._ as a function of SPDC pump intensity.



The EPP detection efficiency of this system (Figure 1a) was determined. As a reference for EPP detection efficiency, the detection efficiency of EPPs transmitted through a cell containing DMSO was determined. *Cτ* values were measured at each SPDC pump light intensity, which was varied by changing the transmittance of VND_1_. Figure 4 shows an example of a histogram obtained from the coincidence measurement when the SPDC pump light intensity was 34 μW. Here, a peak appeared at 43 ns in the measured histogram (Figure 4a). _*A*_ *R*_solv._ (Figure 4b) was calculated from *Cτ* in the off‐peak delay time region (0–39 ns). Therefore, _*E*_ *R*_solv._ was obtained by subtracting _*N*_ *R* and _*A*_ *R*_solv._ from _*T*_ *R*_solv._ as follows:

(12)

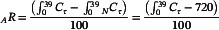






(13)

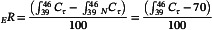




The linear proportionality of _*A*_ *R*_solv._ to the SPDC pump light intensity indicated that _*A*_ *R*_solv._ originated from the SPDC photons. Next, in Figure 4c, the obtained _*E*_ *R*_solv._ value was plotted as a function of the SPDC pump light intensity. The linear dependence of _*E*_ *R*_solv._ on the SPDC pump, light intensity indicates that _*E*_ *R*_solv._ originates from the SPDC photons. From the slope in Figure 4c, the EPP detection efficiency of this system was determined to be 58 pairs·μW^–1^·s^–1^.

### 2.9 Dependence of _*E*_ *R* and _*A*_ *R* on the Transmittance of VND_1_ and VND_2_

**FIGURE 5:**
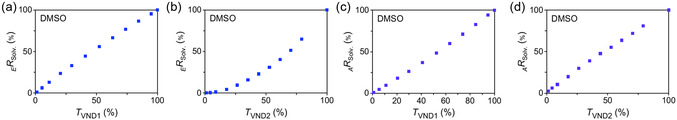
Dependence of the normalized _*E*_ *R*_solv._ values and _*A*_ *R*_solv._ values on the transmittance of VND_1_ and VND_2_. (a,b) Normalized _*E*_ *R*_solv._ value versus transmittance of (a) VND_1_ and (b) VND_2_. (c,d) Normalized _*A*_ *R*_solv._ value versus transmittance of (c) VND_1_ and (d) VND_2_.

To confirm that _*E*_ *R* originates from EPP events and that _*A*_ *R* originates from EUP events, we examined the dependence of *Cτ* on the VND_1_ transmittance, which controls the SPDC pump intensity, and on the VND_2_ transmittance, which attenuates the intensity of the generated SPDC photons after the crystal. As in the previous section, DMSO was used as the solvent. *Cτ* values were measured at each VND_1_ transmittance. Because ϕ of EPP shows a linear dependence on _*E*_ *R*_solv._ value, the normalized _*E*_ *R*_solv._ values (percentage of the _*E*_ *R*_solv._ value at the maximum transmittance) were plotted as a function of the VND_1_ transmittance (Figure 5a). The linear dependence of _*E*_ *R*_solv._ (i.e., ϕ) on the VND_1_ transmittance provides the first evidence that the detected signals originate from SPDC photons. Next, *Cτ* values were measured at each VND_2_ transmittance. Normalized _*E*_ *R*_solv._ values were plotted as a function of the VND_2_ transmittance (Figure 5b). The quadratic dependence of _*E*_ *R*_solv._ (i.e., ϕ) on the VND_2_ transmittance provides the second evidence that the coincidence counts originate from EPPs.

In contrast, the _*A*_ *R*_solv._ value exhibited a linear dependence on the transmittance of both VND_1_ and VND_2_ (Figure 5c,d) indicating that _*A*_ *R*_solv._ originates from counts in a delay‐time region where detections at SPCM‐1 and SPCM‐2 are uncorrelated (i.e., accidental coincidences). When the VND_1_ transmittance is varied, both ϕ of EPP at the BS and $\phi^{\prime }$ of EUP at the BS scale linearly with the VND_1_ transmittance; consequently, both the _*E*_ *R*_solv._ value, which represents EPP events, and the _*A*_ *R*_solv._ value, which represents EUP events, scales linearly with the VND_1_ transmittance. When the VND_2_ transmittance is varied, ϕ of EPP at the BS scales with the square of the VND_2_ transmittance, whereas $\phi^{\prime }$ of EUP at the BS scales linearly with the VND_2_ transmittance. Accordingly, the _*E*_ *R*_solv._ value also shows a quadratic dependence on the VND_2_ transmittance, whereas _*A*_ *R*_solv._ value scales linearly with the VND_2_ transmittance. Thus, in this system, _*E*_ *R*_solv._ and _*A*_ *R*_solv._ can be evaluated independently by separating the correlated peak (true‐pair events) from the uncorrelated background (accidental coincidences) in the coincidence histograms (Figure 4a) acquired under various conditions.

By acquiring coincidence histograms over a delay‐time range of 0–100 ns at count rates within the linear‐response regime of the detectors (≤1 × 10^6^ counts·s^–1^), the transmitted EPPs and EUPs can be quantified independently. This capability enables the simultaneous evaluation of _*E*_ *R* and _*A*_ *R*, which are difficult to disentangle using conventional EPP‐transmittance measurements [30, 31, 32, 33] because both contributions show linear dependences.

### 2.10 Measurement of and 

The cells for the sample solution and solvent were mounted on the stage; this enabled the exchange of the cells on the optical path without opening the black box. To minimize the influence of temporal fluctuations in the light‐source intensity on the measurement results, 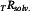 and 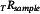 were measured as a paired set at each SPDC pump light intensity for a total of 11 different intensities.

### 2.11 Measurement of Linear Loss

The linear loss is defined as the difference in the stationary transmittance (*T*) between the cell containing the sample solution and the cell containing the solvent. This loss can primarily be attributed to two factors. First, the transmittance can differ between cells because of cell‐to‐cell variations (e.g., scattering differences). Second, weak absorption can arise from the long‐wavelength absorption tail of the sample solution or from the formation of solute aggregates in the solution. _*A*_ *R* originates from EUPs. Here, 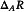 is defined as 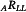.



(14)

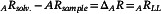




These LL are unrelated to the ETPA, affect the transmittance of both the EPP and the EUP in a similar manner, and can be calculated using _*A*_ *R* as follows:




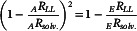







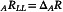






(15)

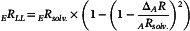




### 2.12 Baseline Measurement

The measurement error was estimated using two different cells containing DMSO (Solv. and Sample) as a paired set. The *Slope* was calculated as follows:



(16)

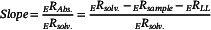




**FIGURE 6:**
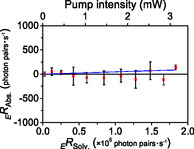
Baseline measurement results.

The average of three measurements (Figure S11 in the Supporting Information) gave Slope=0.0005±0.0002 (Figure 6). This result indicates that this system can detect a transmittance difference of approximately 0.1% or larger (details are described in Section S3 of the Supporting Information).

### 2.13 Measurement of the CTPA Cross‐Section ($\delta_{C}^{\left(2\right)}$)

$\delta_{C}^{\left(2\right)}$ represents the efficiency of CTPA induced by uncorrelated photons (classical photons) and was measured as follows. $\delta_{C}^{\left(2\right)}$ of the sample solution was measured by the Z‐scan method using a Ti:sapphire oscillator (TSUNAMI, Spectra‐Physics Inc.) with a central wavelength of 814 nm, a repetition rate of 80 MHz, and a pulse duration of 100 fs. The experimental conditions other than the light source, specifically the focused area and the interaction volume, were kept the same as those used in the ETPA measurements (A = 2.9 × 10^–5^ cm^2^, V = 2.9 × 10^–6^ cm^3^). $\phi^{\prime }$ at the focal spot was 4.8 × 10^26^ $\text{photons}\cdot s^{-1}\cdot {\mathrm{cm}}^{-2}$.

### 2.14 Measurement of the ETPA Cross‐Section ($\sigma_{E}$)

The detailed data are shown in Section S4 of the Supporting Information. Figure S12 shows representative coincidence histograms obtained from the coincidence measurements when the SPDC pump light intensity was 34 μW. _*A*_ *R*_*4‐AmAB*_ was calculated from *C*_*τ*_ in the off‐peak delay‐time region using Equation (12), and the resulting _*A*_ *R*_*4‐AmAB*_ values were plotted as a function of the SPDC pump light intensity (Figure S13). _*E*_ *R*_*4‐AmAB*_ was then calculated using Equation (13), and the resulting _*E*_ *R*_*4‐AmAB*_ values were plotted as a function of the SPDC pump light intensity (Figure S14). (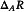 (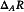
=_*A*_ *R*_*solv.*_–_*A*_ *R*_*sample*_) and 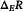 (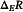
=_*E*_ *R*_*solv.*_–_*E*_ *R*_*sample*_) were obtained from three independent measurements at 1 and 5 mol·L^–1^ (Figures S15 and S16 for 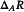; Figures S18 and S19 for 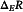). For each concentration, the three datasets were averaged to give the mean 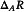 and 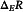 values (Figures S17 and S20). For both samples, 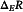 was positive (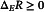; Figure S20).

**FIGURE 7:**
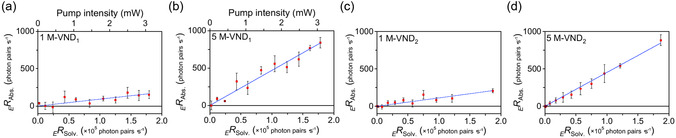
Dependence of _*E*_ *R*_*Abs.*_ value on _*E*_ *R*_*solv.*_ value. (a,b) Transmittance varied using VND_1_. (c,d) Transmittance varied using VND_2_. (a,c) 1 M; (b,d) 5 M.

Next, _*E*_ *R*_*LL*_ was estimated using Equation (15). Furthermore, _*E*_ *R*_*Abs.*_ values were calculated by correcting 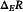 using _*E*_ *R*_*LL*_, and were plotted as a function of the corresponding _*E*_ *R*_*solv.*_ values (Figure 7a,b). The raw datasets from the three independent measurements are shown in Figures S21 for 1 mol·L^–1^ and S22 for 5 mol·L^–1^, and in Table S1. For both samples, _*E*_ *R*_*Abs.*_ was positive (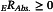) over the entire range of _*E*_ *R*_*solv.*_, indicating absorption of EPPs by the sample. The *Slopes* (Table 1) were determined from Figure 7a,b, and $\sigma_{E}$ was calculated using Equation (7) with the following parameters: solution concentration (c: mol·L^–1^), focused area (A: 2.9 × 10^–5^ cm^2^), interaction volume (V: 2.9 × 10^–6^ cm^3^), and optical path‐length (0.1 cm). The resulting $\sigma_{E}$ values are summarized in Table 2.

**TABLE 1:** Measured slope for both solutions (average of three measurements).

Concentration (mol·L^–1^)	Slope		$\sigma_{E814nm}$(×10^–23^ cm^2^·molecule^–1^)	
	VND_1_	VND_2_	VND_1_	VND_2_
1	0.0009±0.0001	0.0011±0.0001	1.5±0.2	1.6±0.1
5	0.0047±0.0002	0.0045±0.0001	1.8±0.2	1.5±0.0

**TABLE 2:** Measured absorption cross‐sections of 4‐AmAB.

Cross‐sections	4‐AmAB
$\sigma_{E814\mathrm{nm}}$(×10^–23^ cm^2^·molecule^–1^)	1.6±0.1^a^
$\delta_{C407\mathrm{nm}}^{\left(1\right)}$(×10^–23^ cm^2^·molecule^–1^)	1.1 × 10^7^
$\delta_{C814\mathrm{nm}}^{\left(2\right)}$(×10^–50^ cm^4^·s·photon^–1^·molecule^–1^)	17
$\delta_{C814\mathrm{nm}}^{\left(2\right)}\cdot \phi^{'}$(×10^–23^ cm^2^·molecule^–1^)^b^	1.7 × 10^−18^

a Average of four values.

b In the case of $\phi^{\prime }$=1 × 10^8^ (photon·s^‐1^·cm^–2^).

These results were obtained by evaluating the ϕ‐dependence of _*E*_ *R*_Abs._ while varying the VND_1_ transmittance (i.e., the SPDC pump light intensity). Because _*E*_ *R*_Abs._ exhibited a linear dependence on ϕ, the absorption process induced by EPP was identified as a linear process. Many previous ETPA studies have similarly assessed the ϕ‐dependence of _*E*_ *R*_Abs._ by changing the SPDC pump light intensity.

In addition to varying the VND_1_, we also evaluated the ϕ‐dependence of _*E*_ *R*_Abs._ by keeping the SPDC pump light intensity constant at 3.1 mW and varying the VND_2_ transmittance (the detailed data are shown in Section S5 of the Supporting Information). In this measurement scheme, _*A*_ *R*_*solv.*_ (and _*A*_ *R*_*4‐AmAB*_) show a first‐order dependence on the VND_2_ transmittance, whereas _*E*_ *R*_*solv.*_ (and _*E*_ *R*_*4‐AmAB*_) show a second‐order dependence; these quantities were used to calculate _*E*_ *R*_Abs__._ in the same manner as described above. Figure S23 shows the normalized _*A*_ *R*_*solv.*_ (and _*A*_ *R*_*4‐AmAB*_) values plotted as a function of the VND_2_ transmittance, and Figure S24 shows the normalized _*E*_ *R*_*solv.*_ (and _*E*_ *R*_*4‐AmAB*_) values plotted as a function of the VND_2_ transmittance. Using these data, 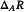 (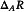
=_*A*_ *R*_*solv.*_–_*A*_ *R*_*sample*_) and 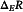 (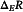
=_*E*_ *R*_*solv.*_–_*E*_ *R*_*sample*_) were calculated at each VND_2_ transmittance, and _*E*_ *R*_Abs._ was obtained using Equations (2) and (15). The resulting plot of _*E*_ *R*_Abs__._ versus _*E*_ *R*_*solv.*_ is shown in Figure 7c,d. The $\sigma_{E}$ values calculated from the slope in Figure 7c,d were comparable to those obtained from Figure 7a,b (Table 1).

In the region where multiphoton excitation is not induced, $\sigma_{E}$ for 4‐AmAB is only approximately 10^7^‐fold lower than $\delta_{C}^{\left(1\right)}$. Thus, $\sigma_{E}$ lies within seven orders of magnitude of $\delta_{C}^{\left(1\right)}$, consistent with theoretical predictions [48]. Moreover, at $\phi^{\prime }$ of ≈10^8^ photons·s^–1^·cm^–2^, $\sigma_{E}$ is approximately 10^18^‐fold larger than $\delta_{C}^{\left(2\right)}$. Whereas CTPA is not measurable unless $\phi^{\prime }$ exceeds 10^26^ photons·s^–1^·cm^–2^, ETPA is measurable even when $\phi^{\prime }$ is around 10^8^ photons·s^–1^·cm^–2^. It should be noted that conventional theories of ETPA [21, 25, 26, 27, 28, 29, 37, 38, 39, 40] presume the simultaneous incidence of two photons on molecules and focus on the efficiency difference due to the presence of entanglement between the two photons, wherein the ETPA efficiency increases by several orders of magnitude. In this study, however, we compare ETPA induced by a low photon‐flux density of 10^8^ photons·s^–1^·cm^–2^ with TPA induced by a pulsed laser attenuated to the same photon‐flux density level as the ETPA. Generally, the probability of two‐photon coincidences in laser light is extremely low, even for an ultrashort pulse. Therefore, the efficiency enhancement observed in this study cannot be explained only by the enhancement due to quantum entanglement between photons, and this point is discussed in the next section.

### 2.15 Considerations for Comparison With Theory

Finally, we discuss an important point that should be taken into consideration when comparing experimental results of ETPA with theoretical predictions. Most conventional theoretical works focus on the presence or absence of quantum entanglement between two photons, in other words, whether the intensity dependence of TPA is linear, $\langle E^{-}E^{-}E^{+}E^{+}\rangle \propto I$, or quadratic, $\langle E^{-}E^{-}E^{+}E^{+}\rangle =\langle E^{-}E^{+}\rangle \langle E^{-}E^{+}\rangle \propto I^{2}$. Even in the recent theories using the two‐photon wavefunction of $\psi (\omega ,\omega^{\prime })$, the key issue is whether $\psi (\omega ,\omega^{\prime })$ can be factorized into a direct product, $\psi (\omega ,\omega^{\prime })=\phi (\omega )\varphi (\omega^{\prime })$, or not. In these cases, the quantum states of photons are treated as Fock states (photon number states), and therefore, the simultaneity of two photons is implicitly assumed because exactly two photons are considered. However, most TPA experiments are performed using a laser, which is theoretically described as a coherent state. As is well known, fully quantum‐mechanical pulsed coherent light is described as [see, e.g., “Quantum Theory of Light”, Loudon]

(17) $$\left|\alpha_{\psi }\right\rangle =\exp(-{\left|\alpha \right|}^{2}/2)(|0\langle +\alpha \int d\omega \psi (\omega ){\hat{a}}^{†}(\omega )|0\rangle +\frac{\alpha^{2}}{2!}\int d\omega \int d\omega^{\prime }\psi \left(\omega \right)\psi \left(\omega^{\prime }\right){\hat{a}}^{†}\left(\omega \right){\hat{a}}^{†}\left(\omega^{\prime }\right)\left|0\right\rangle +\ldots )$$

This state indicates that, for a low intensity of α≪1, the vacuum state |0⟩ is dominant and hence the simultaneity of two photons is inherently quite low even for an ultrashort pulse. In fact, photons in coherent light are randomly generated, following a Poisson distribution, and therefore, two photons are generated fully accidentally. On the other hand, a photon pair generated in SPDC possesses inherent simultaneity, which originates from the squeezed light consisting of an even number of photons. This fact strongly indicates that the low efficiency of CTPA originates from the lack of simultaneity of photons. Thus, ideally, we should consider three types of photon states, namely, an entangled two‐photon state, a disentangled two‐photon state with simultaneity, and a pulsed coherent state, to properly compare the entanglement‐enhanced TPA process with TPA induced by coherent light. This issue will be addressed in future work. Thus, the observed enhancement in the effective two‐photon excitation efficiency in this work can be regarded as reasonable when the difference in two‐photon simultaneity under low photon‐flux conditions is taken into account.

## 3 Conclusion

In this study, we constructed an ETPA measurement system using time‐entangled photon pairs generated by SPDC with a CW 407‐nm laser. By measuring the time correlation between the two photons transmitted through a sample solution and separating correlated from uncorrelated coincidence counts, we evaluated the ETPA cross‐section, $\sigma_{E},$ of 4‐aminoazobenzene, which is a small organic molecule with extremely low one‐photon absorption and negligible scattering in the EPP wavelength range (750–850 nm). The ETPA cross‐section is approximately 10^7^‐fold lower than the corresponding one‐photon absorption cross‐section. In addition, the ETPA cross‐section is approximately 10^18^‐fold larger than the CTPA cross‐section. Whereas CTPA is not measurable unless $\phi^{\prime }$ exceeds 10^26^ photons·s^–1^·cm^–2^, ETPA could be measured even when $\phi^{\prime }$ is around 10^8^ photons·s^–1^·cm^–2^. Overall, this time‐correlation–based approach provides a robust protocol for distinguishing linear loss from ETPA and for quantifying $\sigma_{E}$ at photon‐counting–level flux densities. Looking ahead, brighter entangled‐photon sources based on SPDC in periodically poled crystals (e.g., PPKTP) should increase the photon‐pair rate at the sample. This improvement is expected to shorten the acquisition time and facilitate ETPA measurements for weaker absorbers.

## Supporting Information

Additional supporting information can be found online in the Supporting Information section.

## Funding

This study was supported by Japan Society for the Promotion of Science (22KJ1395, 25K21714); Kanagawa University (2023A004).

## Conflicts of Interest

The authors declare no conflicts of interest.

## Supporting information

Supplementary Material

## Data Availability

The data that support the findings of this study are available from the corresponding author upon reasonable request.
