# Supplementary material for: Measurement of the Entangled Two‐Photon Absorption Cross‐Sections of 4‐Aminoazobenzene
Source: Chemphyschem. 2026 May 11;27(9):e70403. doi: 10.1002/cphc.70403 (PMC13184008; doi:10.1002/cphc.70403)
Supplement: Supplementary file 1 — Supplementary Material [file CPHC-27-e70403-s001.pdf]

Supporting Information

Measurement of the Entangled Two-Photon Absorption Cross-Sections of 4-Aminoazobenzene

Sena Hashimoto,[a,b*] Hisaki Oka,[a*] Izumi Iwakura,[a] Tomoyuki Horikiri,[c]

[a] *Kanagawa University, 3-27-1 Rokkakubashi, Yokohama 221-8686, Japan.*
[b] *Society for the Promotion of Science (JSPS), Kojimachi Business Center Building, 5-3-1 Kojimachi, Chiyoda-ku, Tokyo 102-0083, Japan.*
[c] *Yokohama National University, 79-5 Tokiwadai, Yokohama 240-8501, Japan.*

Contents

Section S1. Sample selection S2
Section S2. Construction of the ETPA measurement system S5
Section S3. Baseline measurement S7
Section S4. ETPA measurement results varying VND_1 transmittance S9
Section S5. ETPA measurement results varying VND_2 transmittance S13

Section S1. Sample selection

Commercially available Rhodamine B (RhB) is often used for the ETPA measurements. In many studies, RhB (TOKYO CHEMICAL INDUSTRY Co., Ltd.) in methanol (MeOH, spectrophotometric grade; KANTO CHEMICAL Co., Inc.) solution has been examined over the concentration range 0.05~110 $\times 10^{-3}$ mol·L^{-1} using a screw-capped silica-quartz cell with an internal path-length of 10 mm (S15-IR-10, GL Science Inc.). We measured the UV–visible stationary transmittance spectrum of RhB in methanol at 110$\times 10^{-3}$ mol·L^{-1} (Figure S1a) using a UV–visible spectrophotometer (V-650, JASCO Inc.). The weak absorption feature around 800 nm observed at the higher concentration originates from the long-wavelength absorption tail of RhB.

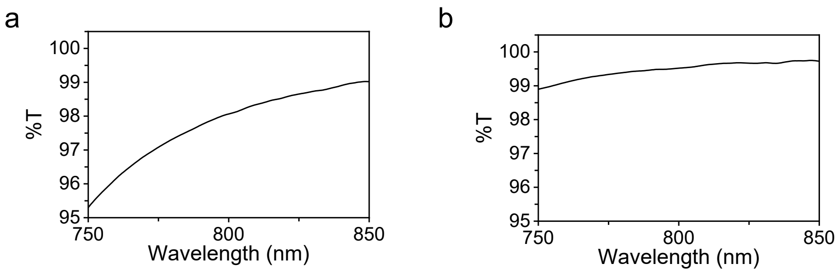

Figure S1. Stationary transmittance spectra of RhB in methanol: (a) 110$\times 10^{-3}$ mol·L^{-1}, 10-mm path-length, (b)100$\times 10^{-3}$ mol·L^{-1}, 1-mm path-length).

As shown in Figure S1a, RhB in methanol solition exhibits steady-state absorption in the wavelength region of the EPPs used in our measurements. Because our setup employs a 1-mm optical path-length solution cell, we additionally measured the stationary transmittance spectrum of RhB in MeOH (100$\times 10^{-3}$ mol·L^{-1}) solution using a 1-mm cell (Figure. S1b). All solutions were stored in a screw-capped silica-quartz cells with an internal path-length of 1 mm (S15-IR-1, GL Science Inc.) for the stationary absorption, CTPA, and ETPA measurements. As shown in Figure S1b, the RhB in MeOH (100$\times 10^{-3}$ mol·L^{-1}) solution exhibits measurable absorption at the ~1% level at 750 nm (%T = 99%). We then attempted ETPA measurements using this solution.

Methanol and the RhB methanol solutions were measured. Figure S2 shows a representative histogram obtained from the coincidence measurements at the SPDC pump light intensity of 34 µW. ${}_{A}R_{MeOH}$ (${}_{A}R_{RhB}$) was calculated from C_t in the off-peak delay time region using Equation (12), and the resulting ${}_{A}R_{RhB}$ values were plotted as a function of the SPDC pump light intensity (Figure S3). ${}_{E}R_{MeOH}$ (${}_{E}R_{RhB}$) was then calculated using Equation (13). The resulting ${}_{E}R_{MeOH}$ and ${}_{E}R_{RhB}$ values were plotted as a function of the SPDC pump light intensity Figure S4).

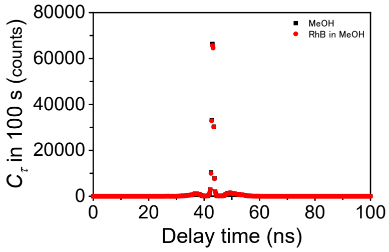

Figure S2. Results from the coincidence counting measurement for EPPs transmitted through the cell containing methanol (black line) and the RhB in methanol (red line) at an SPDC pump intensity of 34 µW.

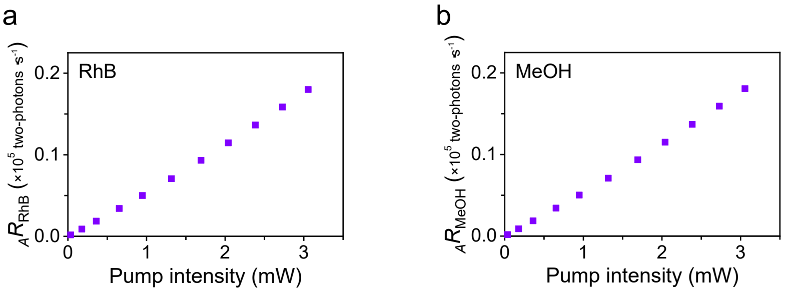

Figure S3. Dependence of ${}_A R$ value on the SPDC pump light intensity: a: Methanol and b: RhB in methanol.

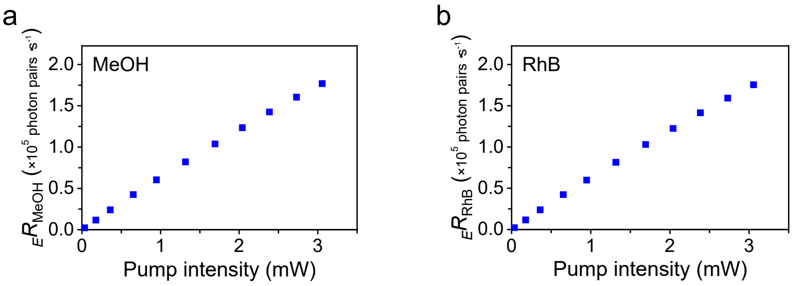

Figure S4. Dependence of ${}_E R$ value on the SPDC pump light intensity: a: Methanol and b: RhB in methanol.

The calculated $\Delta_A R$ ($\Delta_A R = {}_A R_{MeOH} - {}_A R_{RhB}$) and $\Delta_E R$ ($\Delta_E R = {}_E R_{MeOH} - {}_E R_{RhB}$) values were positive, as shown in Figures S5a and S5b. As shown in Figure S5a, the Slope of $\Delta_A R$ was 0.0043 ± 0.0008, corresponding to a transmittance of 99.57 ± 0.08%, which is in good agreement with the transmittance at 800 nm in the spectrum shown in Figure S1b (99.53%). Next, ${}_E R_{Abs.}$ value (${}_E R_{Abs.} = \Delta_E R - {}_E R_{LL} = \Delta_E R - {}_E R_{MeOH} \times (\Delta_A R / {}_A R_{MeOH})$) at each SPDC pump light intensity was plotted as a function of the corresponding ${}_E R_{MeOH}$ value (Figure S5c). ${}_E R_{Abs.}$ was approximately 0 over the entire range of ${}_E R_{MeOH}$, indicating that no measurable absorption of EPPs by the sample occurred. These results were consistent with Ref. 41 and suggests that ETPA might not be measurable if the stationary absorption was greater than the ETPA.

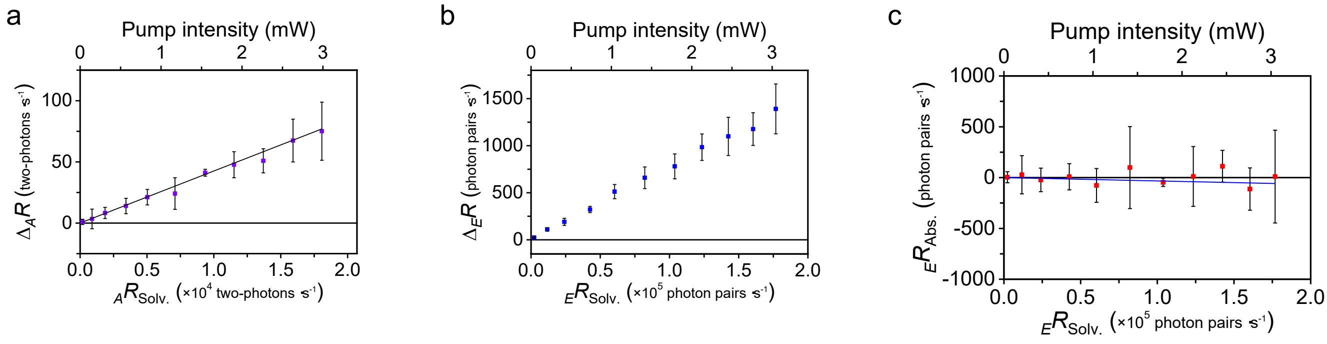

Figure S5. Results for the measurement of RhB in methanol: a: Dependence of $\Delta_A R$ on ${}_A R_{solv.}$, b: dependence of $\Delta_E R$ on ${}_E R_{solv.}$ and c: dependence of ${}_E R_{Abs.}$ on ${}_E R_{solv.}$

Accordingly, we focused on 4-aminoazobenzene (4-AmAB), a small organic molecule exhibiting extremely low linear absorption and light scattering in the EPP wavelength range (750–850 nm). 4-AmAB (TOKYO CHEMICAL INDUSTRY Co., Ltd.) was dissolved in dimethyl sulfoxide (DMSO, spectrophotometric grade; KANTO CHEMICAL Co., Inc.) to prepare solutions with concentrations of 1 and 5 mol·L^{-1}.

Section S2. Construction of the ETPA measurement system

The ETPA measurement system (Figure 1a) was constructed using a CW laser light with a central wavelength of 407 nm as the pump source for SPDC. The SPDC pump light intensity was adjusted by controlling the transmittance of a variable neutral density filter (VND_1). The polarization was then set to horizontal using a half-wave plate (HWP) followed by a polarizing beam splitter (PBS). EPPs were generated via SPDC in a nonlinear crystal (β-BaB_2O_4, BBO; 0.4 mm thick, Type-I, θ = 29.2°, φ = 90°, EKSMA OPTICS) by focusing the pump beam with a plano-convex lens (L_3; f = 50 mm). The generated EPPs were collimated using a plano-convex lens (L_4; f = 50 mm) and then reflected by a dichroic mirror (DM). The residual 407-nm light that was not converted by SPDC and was transmitted through the DM was measured using a power metre (PM). The SPDC pump light intensity incident on the BBO was estimated by correcting for losses due to transmission through the optical components (BBO, L_4, DM) using the actual measured transmittance. Dispersion was further compensated using a chirped mirror pair (CM; DCMP-175, Thorlabs Inc.) and a quartz block (Q).

After the 407-nm light passes through the BBO, the optical axis of the residual pump light coincides with that of the EPP. Before entering the black box that covers the sample cells and detector, the intensity of the 407-nm light is reduced to 1×10^{-6} times the incident intensity on the BBO, primarily due to the wavelength-dependent characteristics of the DMs. However, even after being reflected by the four DMs, the intensity of the 407-nm light at this point remains 4 to 5 orders of magnitude higher than that of the EPP at the same point. Therefore, to further attenuate the intensity of the 407-nm light entering the black box, three interference filters that transmit the EPP wavelength (F_1; two long-pass filters with a cut-on wavelength of 750 nm and one short-pass filter with a cut-off wavelength of 850 nm) were used as the black-box window, providing an additional attenuation of ≈15 orders of magnitude for intensity of the 407-nm light. The EPP (beam diameter: 1.2 mm) entering the black box was focused into a 1-mm path-length sample cell (S; S15-IR-1, GL Science, Inc.) using a plano-convex lens (L_5; f = 50 mm). The transmitted light from the sample cell was collimated with another plano-convex lens (L_6; f = 75 mm). The same wavelength-range filters used for the black box window were then inserted (F_2; one long-pass filter with a cut-on wavelength of 750 nm and one short-pass filter with a cut-off wavelength of 850 nm) to block light from the sample, such as fluorescence, whose wavelength range differs from that of the EPP. After dividing the transmitted light from the sample cell with a beam splitter (BS) into two parts at a 1:1 intensity ratio, the light was then focused onto single-photon counters (SPCM; SPCM-AQR-13; Excelitas Technologies Corp.) using plano-convex lenses (L_7; f = 25 mm). Because L_6 has a longer focal length than L_5, the beam diameter of the transmitted light from the sample cell was expanded compared with the case in which two lenses with equal focal lengths are used. By focusing the beam with L_7 to a spot size sufficiently smaller than the sensor diameter of the SPCM, we eliminated the changes in the coupling efficiency to the SPCM caused by shifts in the optical axis due to movement of the sample cell. A time-correlated single-photon counting board (TCSPC; TimeHarp 260 Nano, PicoQuant GmbH) was used to measure the two-photon coincidence counts, C [two-photons], i.e., the number of detected two-photon events.[47] The time difference between the detection of a photon by SPCM-1 and the detection of a photon by SPCM-2 is the delay time (τ). The number of the single-photons detected by SPCM-2 at each delay time after a single-photon was detected by SPCM-1 ($C\tau$) was measured. After transmission through F_1, the light was divided into two parts using a beam splitter: the reflected light was focused onto SPCM-1, whereas the transmitted light passed through a monochromator and was focused onto SPCM-2. The EPP spectrum (Figure 1b) was obtained by measuring C by changing the wavelength of the monochromator from 700 nm to 900 nm. The intensity of the generated EPP was adjusted by rotating VND_2 to set the transmittance to the desired value. The focused area on the sample (A) was 2.9×10^{-5} cm^2 (see the next paragraph for details); additionally, by considering the optical path-length (0.1 cm), the interaction volume (V) was calculated to be 2.9×10^{-6} cm^3. For example, ϕ at the focal spot was 1.5×10^8 photon pairs $\cdot\ s^{-1} \cdot cm^{-2}$ when the SPDC pump light intensity was 34 μW.

For both the ETPA and CTPA measurement systems, the beam diameter before focusing on the sample was measured

using a two-dimensional sensor, and the focused area was calculated from this diameter and the focal length of the lens that focused the light on the sample. In the CTPA system, the pre-focusing beam diameter was adjusted using a lens pair to match that of the ETPA system, resulting in an estimated focused area of 2.9×10^{-5} cm^2.

Section S3. Baseline measurement

DMSO-1(Solv.) and DMSO-2(Sample) were measured. Figure S6 shows a representative histogram obtained from the coincidence measurements at an SPDC pump light intensity of 34 μW. ${}_{A}R_{DMSO\text{-}1}$ (${}_{A}R_{DMSO\text{-}2}$) was calculated from C_τ in the off-peak delay time region using Equation (12). The obtained ${}_{A}R_{DMSO\text{-}1}$ and ${}_{A}R_{DMSO\text{-}2}$ values were plotted as a function of the SPDC pump light intensity (Figure S7). Although DMSO has no absorption in the EPP wavelength region, the ${}_{A}R_{DMSO}$ values differed between cells, indicating cell-to-cell variations in scattering and reflection.

${}_{E}R_{DMSO\text{-}1}$ (${}_{E}R_{DMSO\text{-}2}$) was then calculated using Equation (13). The calculated ${}_{E}R_{DMSO\text{-}1}$ and ${}_{E}R_{DMSO\text{-}2}$ values were plotted as a function of the SPDC pump light intensity (Figure S8). The resulting $\Delta_{A}R$ ($\Delta_{A}R={}_{A}R_{MeCN\text{-}1}-{}_{A}R_{MeCN\text{-}2}$) and $\Delta_{E}R$ ($\Delta_{E}R={}_{E}R_{MeCN\text{-}1}-{}_{E}R_{MeCN\text{-}2}$) values were positive, as shown in Figures S9 and S10. Because scattering and reflection affect ${}_{A}R$ and ${}_{E}R$ in the same manner, we corrected for linear loss using ${}_{E}R_{LL}=\Delta_{E}R\times$ ($\Delta_{A}R/{}_{A}R_{DMSO\text{-}1}$). After this correction, the slope became approximately zero (Figure S11 and Figure 7). These results indicated that the effects of scattering and reflection were effectively cancelled between ${}_{A}R$ and ${}_{E}R$.

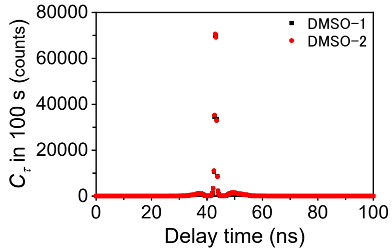

Figure S6. Results from the coincidence counting measurement for the EPPs transmitted through the cell containing DMSO-1 (black square) and DMSO-2 (red square) at an SPDC pump intensity of 34 μW.

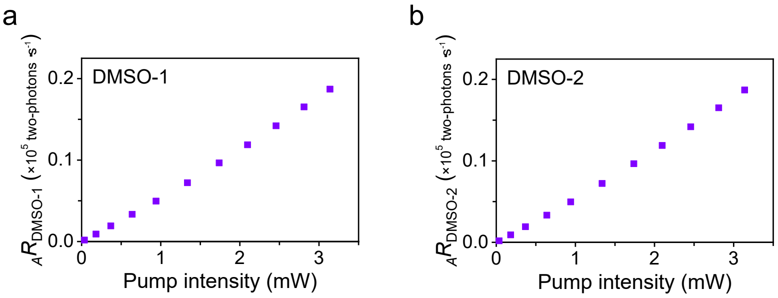

Figure S7. Dependence of ${}_{A}R$ value on the SPDC pump light intensity: (a) DMSO-1, and (b) DMSO-2.

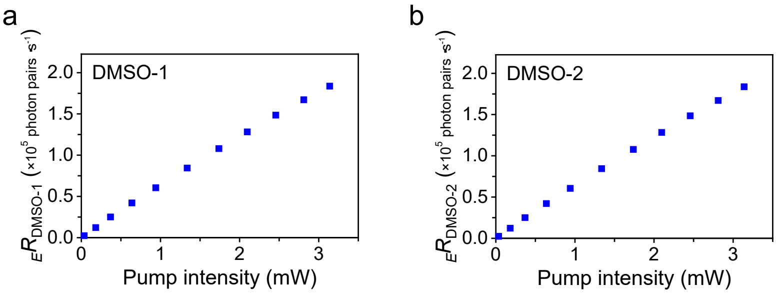

Figure S8. Dependence of ${}_{E}R$ value on the SPDC pump light intensity: (a) DMSO-1, and (b) DMSO-2.

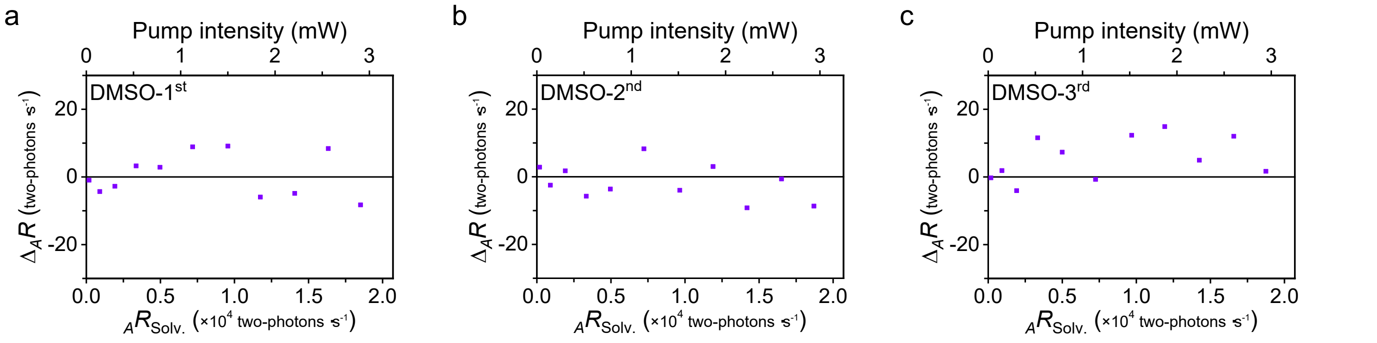

Figure S9. Results of baseline measurements. Dependence of $\Delta_A R$ value on $_A R_{solv.}$ value for three measurements: (a) first, (b) second, and (c) third.

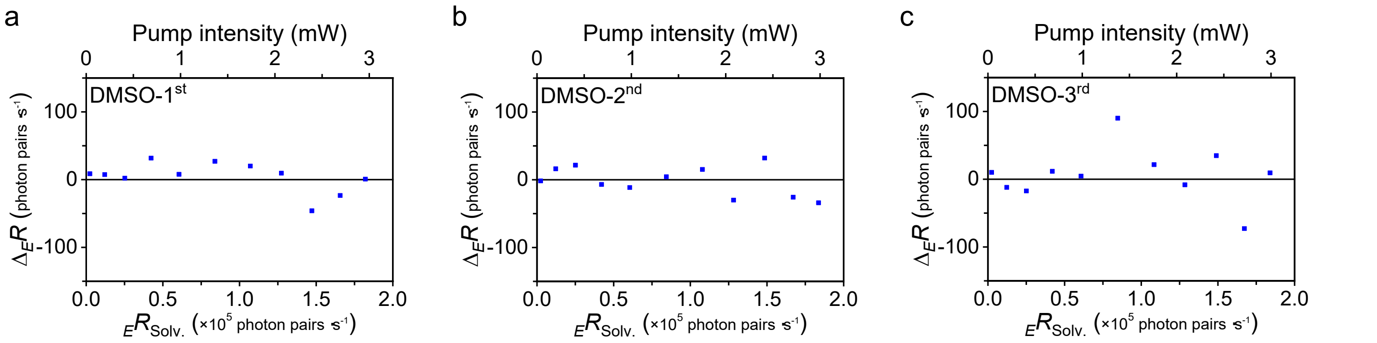

Figure S10. Results of baseline measurements. Dependence of $\Delta_E R$ value on $_E R_{solv.}$ value for three measurements: (a) first, (b) second, and (c) third.

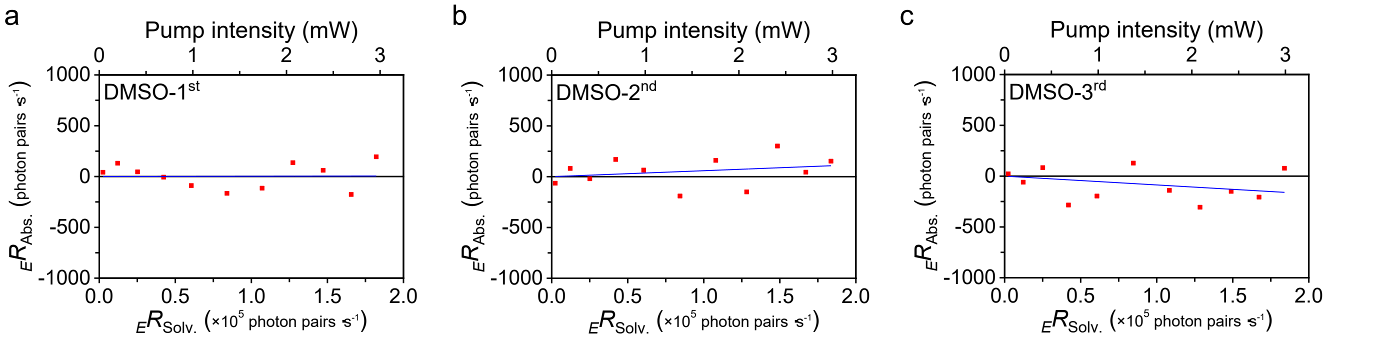

Figure S11. Baseline measurement results for three measurements: (a) first, (b) second, and (c) third.

Section S4. ETPA measurement results varying VND_1 transmittance

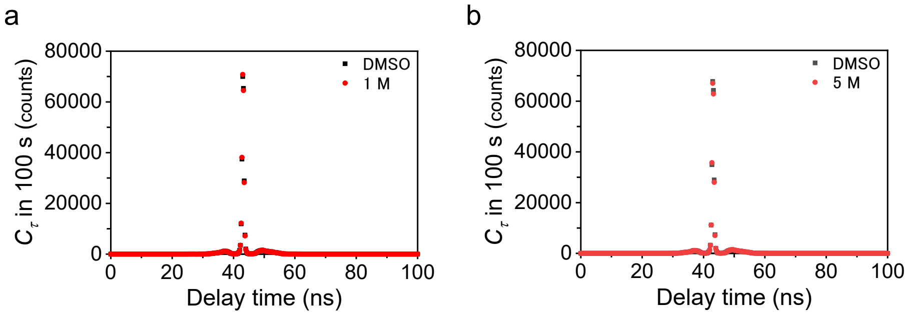

Figure S12. Results from the coincidence counting measurement for the EPP transmitted through the cell containing DMSO (black square) and 4-Aminoazobenzene in DMSO (red circle) at an SPDC pump intensity of 34 μW: (a) 1 M and (b) 5 M.

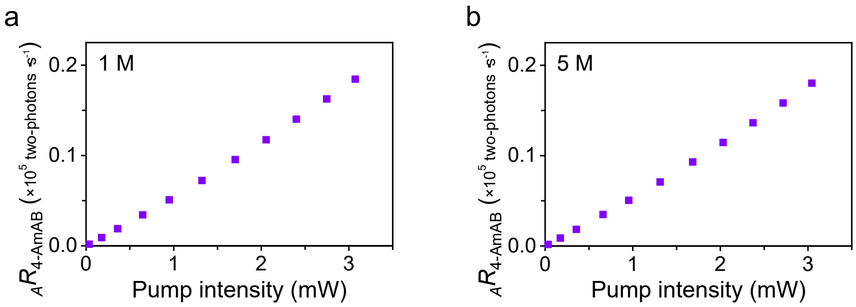

Figure S13. Dependence of $_{A}R_{4\text{-}AmAB}$ on the SPDC pump light intensity: (a) 1 M and (b) 5 M.

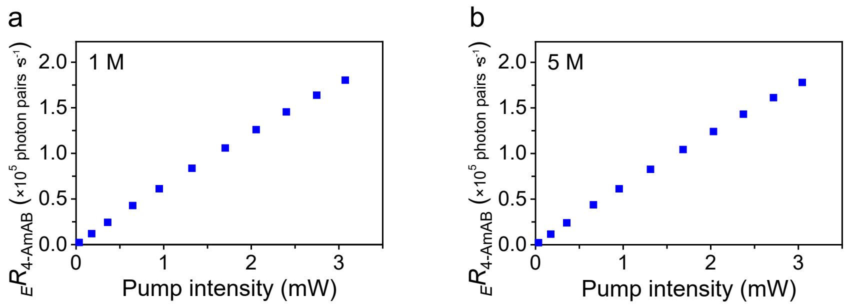

Figure S14. Dependence of $_{E}R_{4\text{-}AmAB}$ value on the SPDC pump light intensity: (a) 1 M and (b) 5 M.

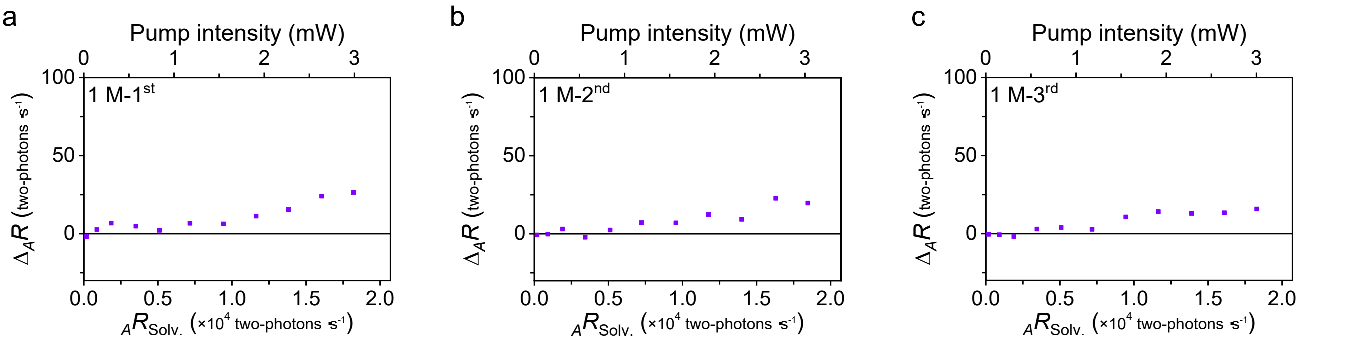

Figure S15. Results of 4-AmAB measurements in DMSO (1 M). Dependence of $\Delta_A R$ value on $_A R_{solv.}$ value for three measurements: (a) first, (b) second, and (c) third.

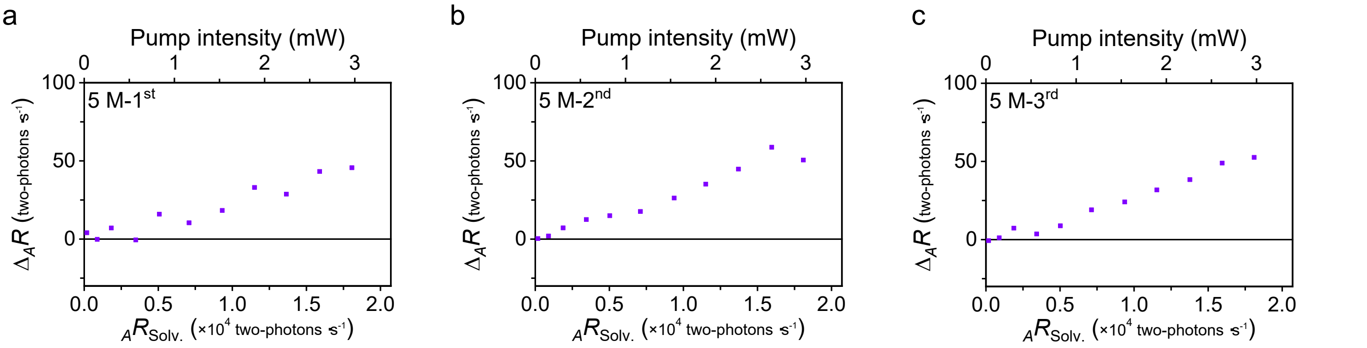

Figure S16. Results of 4-AmAB measurements in DMSO (5 M). Dependence of $\Delta_A R$ value on $_A R_{solv.}$ value for three measurements: (a) first, (b) second, and (c) third.

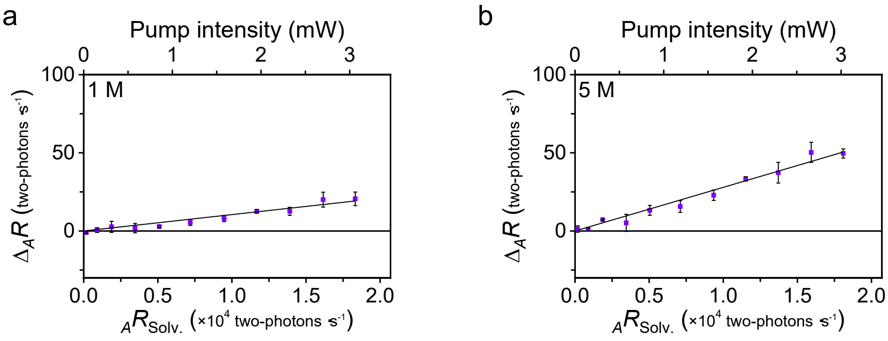

Figure S17. Results of 4-AmAB measurements in DMSO. Dependence of $\Delta_A R$ value on $_A R_{solv.}$ value: (a) 1 M and (b) 5 M.

As shown in Figure S17, the Slope of $\Delta_A R$ was 0.0008 ± 0.0003 for panel (a) and 0.0025 ± 0.0003 for panel (b), corresponding to effective transmittances of 99.92 ± 0.03% and 99.75 ± 0.03 %, respectively (i.e., <0.3% attenuation). These values are in good agreement with the transmittances at 800 nm in the spectrum shown in Figure 2b (99.96% and 99.65%, respectively), confirming that attenuation in the EPP-wavelength range is extremely low even at the highest concentrations.

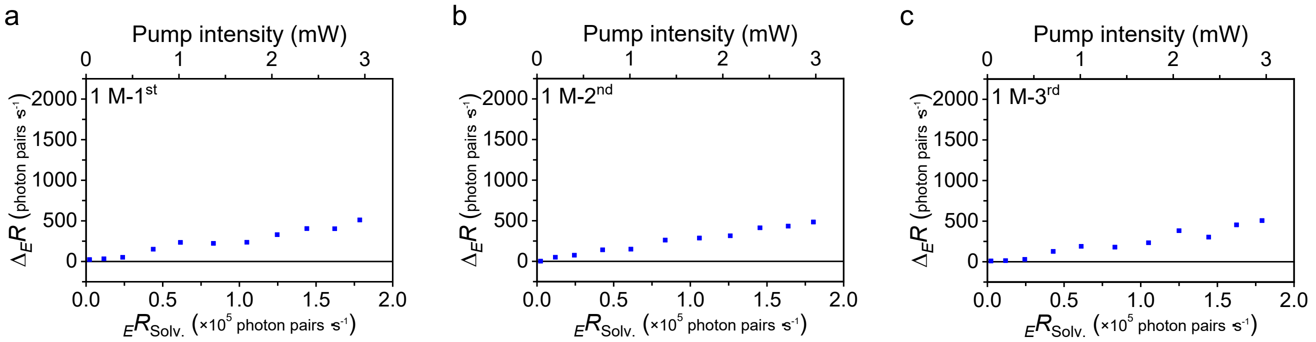

Figure S18 Results of 4-AmAB measurements in DMSO (1 M). Dependence of $\Delta_E R$ value on ${}_ER_{solv.}$ value for three measurements: (a) first, (b) second, and (c) third.

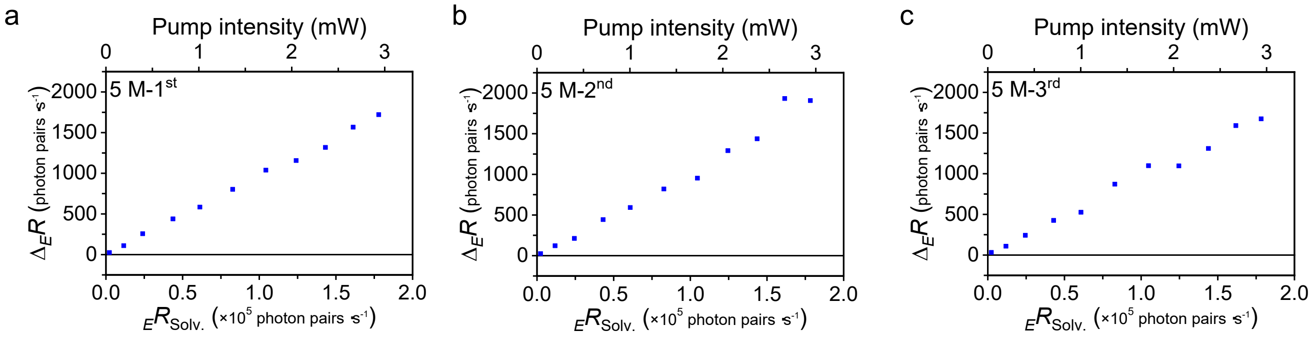

Figure S19. Results of 4-AmAB measurements in DMSO (5 M). Dependence of $\Delta_E R$ value on ${}_ER_{solv.}$ value for three measurements: (a) first, (b) second, and (c) third.

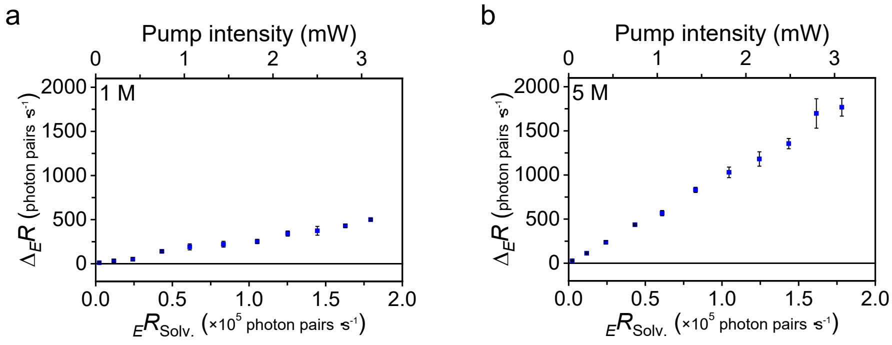

Figure S20. Results of 4-AmAB measurements in DMSO. Dependence of $\Delta_E R$ value on ${}_ER_{solv.}$ value: (a) 1 M and (b) 5 M.

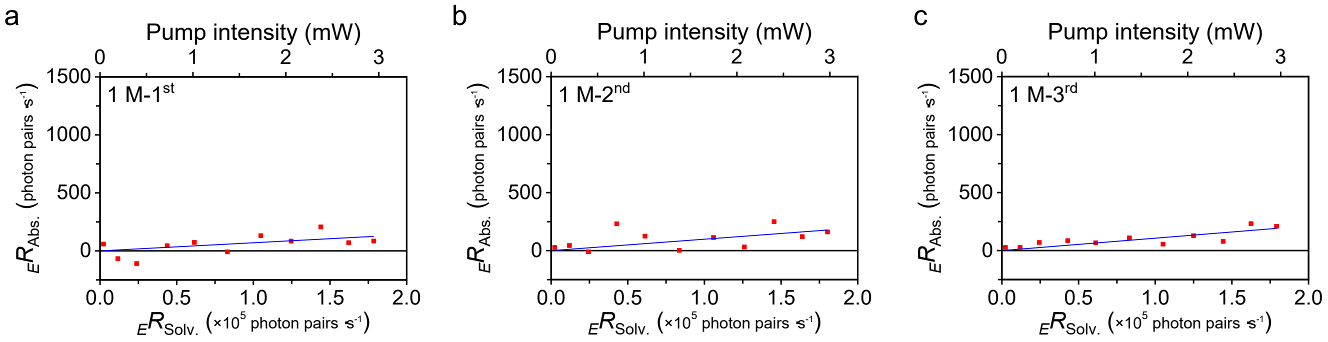

Figure S21. Results of 4-AmAB measurements in DMSO (1 M). Dependence of $_{E}R_{Abs.}$ value on $_{E}R_{solv.}$ value for three measurements: (a) first, (b) second, and (c) third.

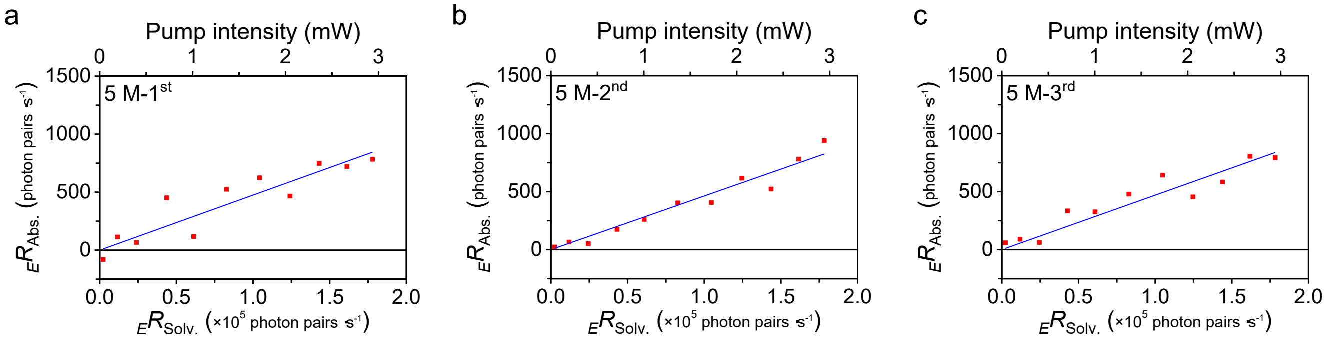

Figure S22. Results of 4-AmAB measurements in DMSO (5M). Dependence of $_{E}R_{Abs.}$ value on $_{E}R_{solv.}$ value for three measurements: (a) first, (b) second, and (c) third.

Table S1. Slope values obtained from the three measurements

	DMSO (solvent)	1M	5M
1	0.0000 ± 0.0004	0.0007 ± 0.0002	0.0048 ± 0.0004
2	0.0006 ± 0.0004	0.0010 ± 0.0002	0.0046 ± 0.0002
3	-0.0009 ± 0.0004	0.0011 ± 0.0001	0.0047 ± 0.0003

Section S5. ETPA measurement results varying VND_2 transmittance

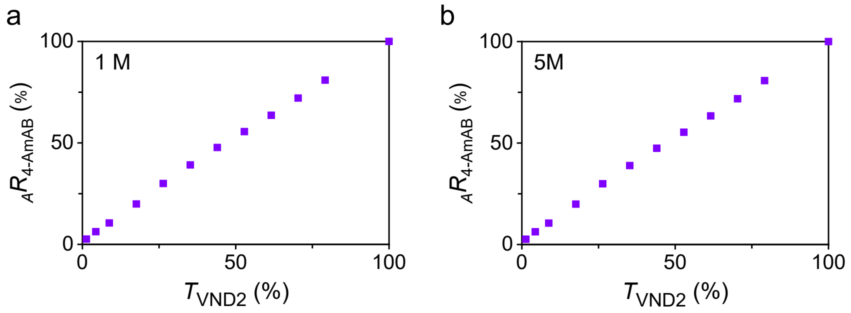

Figure S23. Dependence of the normalized $_{A}R_{4\text{-}AmAB}$ value on VND_2 transmittance: (a) 1 M and (b) 5 M.

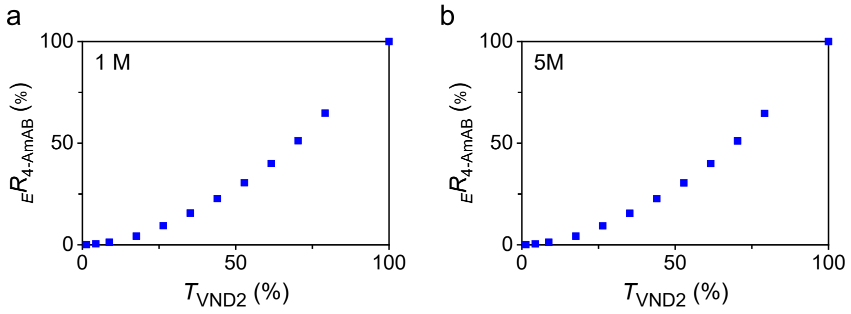

Figure S24. Dependence of the normalized $_{E}R_{4\text{-}AmAB}$ value on VND_2 transmittance: (a) 1 M and (b) 5 M.

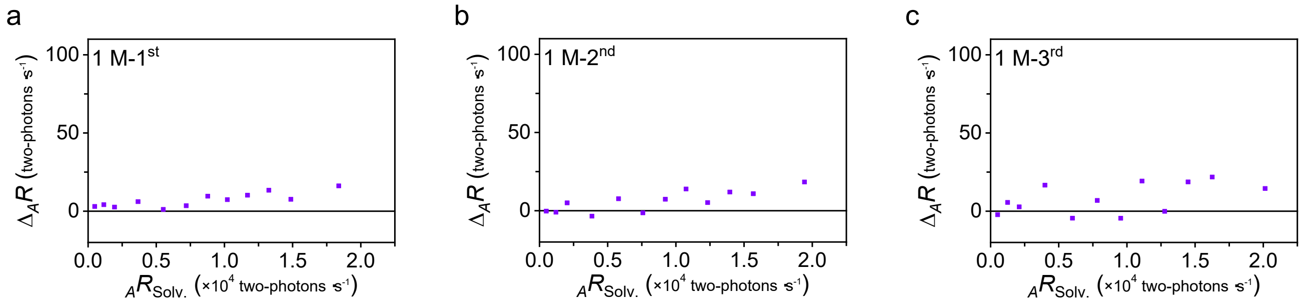

Figure S25. Results of 4-AmAB measurements in DMSO (1 M). Dependence of $\Delta_{A}R$ value on $_{A}R_{solv.}$ value for three measurements: (a) first, (b) second, and (c) third.

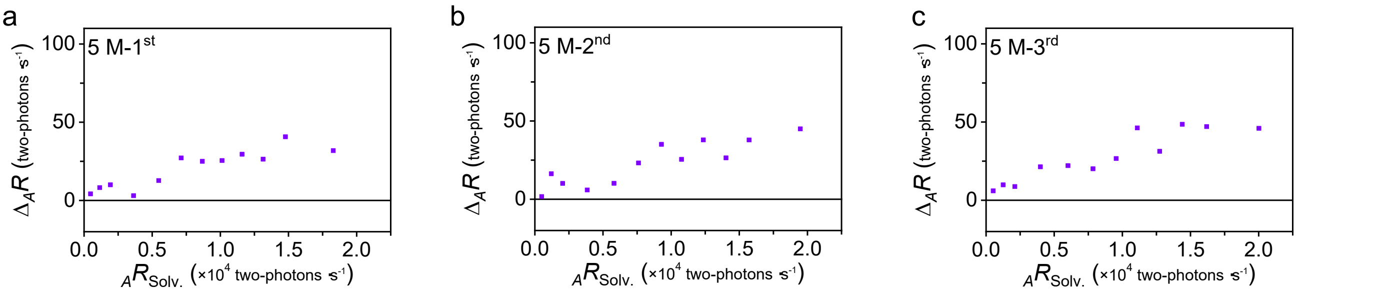

Figure S26. Results of 4-AmAB measurements in DMSO (5 M). Dependence of $\Delta_A R$ value on $_AR_{solv.}$ value for three measurements: (a) first, (b) second, and (c) third.

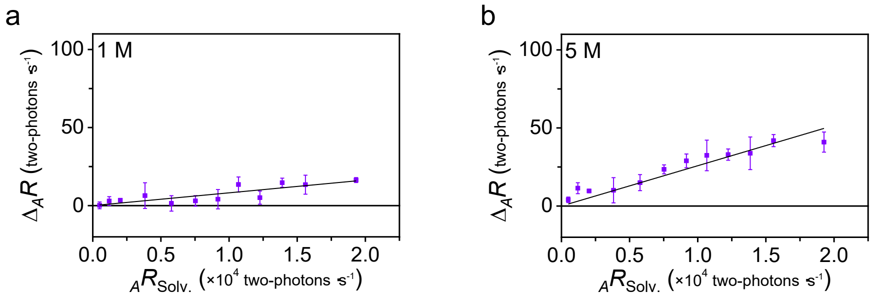

Figure S27. Results of 4-AmAB measurements in DMSO. Dependence of $\Delta_A R$ value on $_AR_{solv.}$ value: (a) 1 M and (b) 5 M.

As shown in Figure S27, the Slope of $\Delta_A R$ was 0.0008 ± 0.0001 for panel (a) and 0.0026 ± 0.0001 for panel (b), corresponding to effective transmittances of 99.92 ± 0.01% and 99.74 ± 0.01 %, respectively (i.e., <0.3% attenuation). These values are in good agreement with the transmittances at 800 nm in the spectrum shown in Figure 2b (99.96% and 99.65%, respectively), confirming that attenuation in the EPP-wavelength range is extremely low even at the highest concentrations.

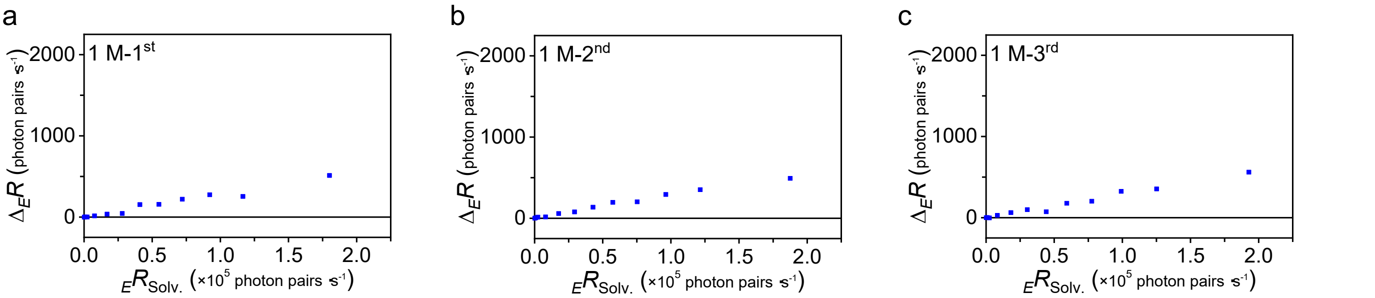

Figure S28 Results of 4-AmAB measurements in DMSO (1 M). Dependence of $\Delta_{E}R$ value on ${}_{E}R_{solv.}$ value for three measurements: (a) first, (b) second, and (c) third.

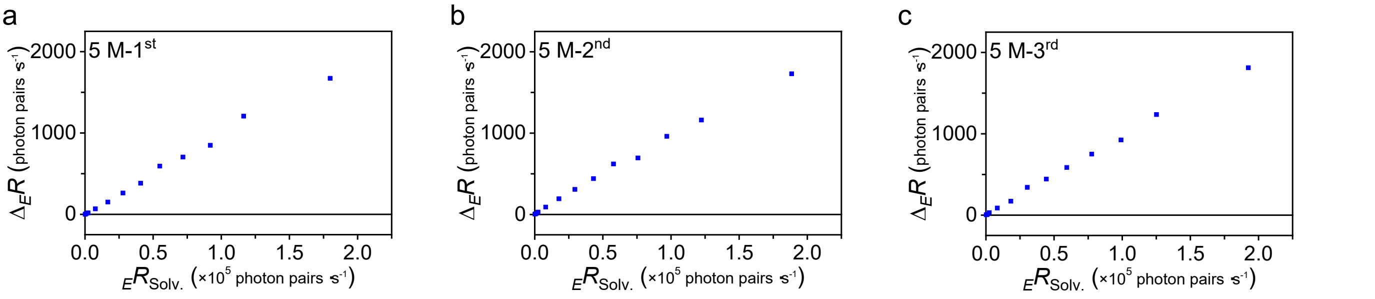

Figure S29 Results of 4-AmAB measurements in DMSO (5 M). Dependence of $\Delta_{E}R$ value on ${}_{E}R_{solv.}$ value for three measurements: (a) first, (b) second, and (c) third.

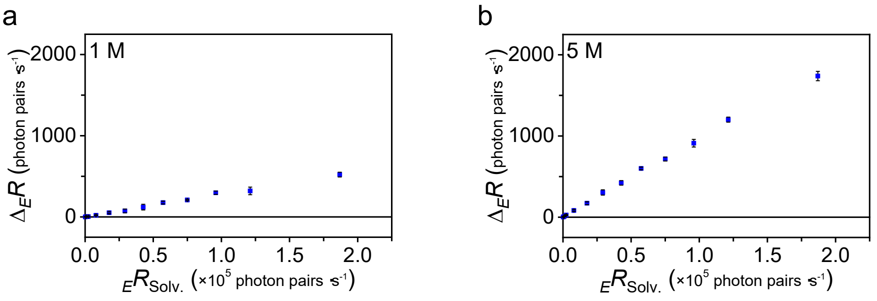

Figure S30. Results of 4-AmAB measurements in DMSO. Dependence of $\Delta_{E}R$ value on ${}_{E}R_{solv.}$ value: (a) 1 M and (b) 5 M.

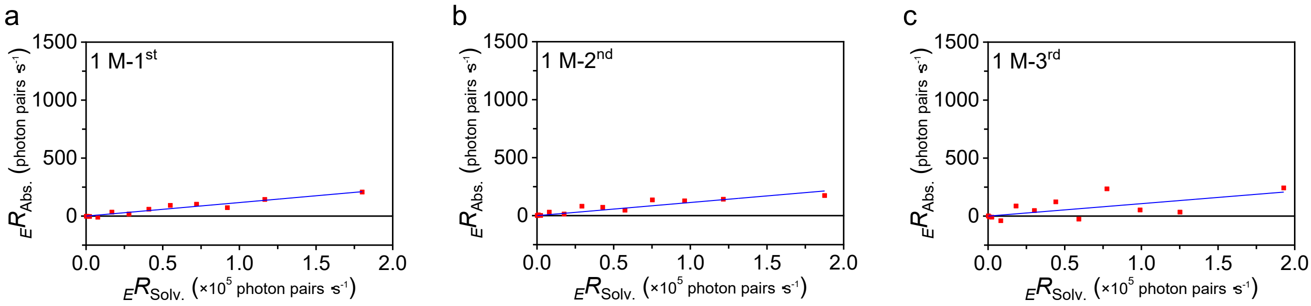

Figure S31. Results of 4-AmAB measurements in DMSO (1 M). Dependence of $_{E}R_{Abs.}$ value on $_{E}R_{solv.}$ value for three measurements: (a) first, (b) second, and (c) third.

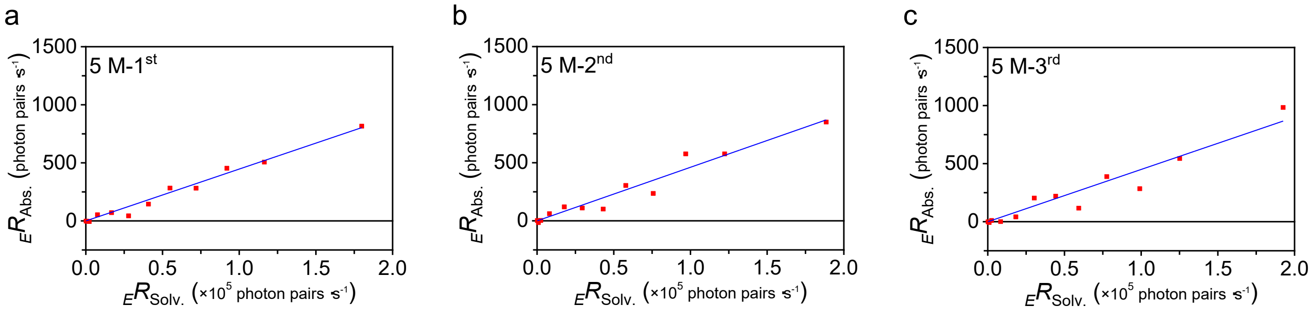

Figure S32. Results of 4-AmAB measurements in DMSO (5 M). Dependence of $_{E}R_{Abs.}$ value on $_{E}R_{solv.}$ value for three measurements: (a) first, (b) second, and (c) third.

Table S2. Slope values obtained from the three measurements

	1 M	5 M
1	0.0012 ± 0.0001	0.0045 ± 0.0001
2	0.0013 ± 0.0001	0.0046 ± 0.0002
3	0.0011 ± 0.0003	0.0045 ± 0.0003
